# Microbiome diversity and reproductive incompatibility induced by the prevalent endosymbiont *Arsenophonus* in two species of African cassava *Bemisia tabaci* whiteflies

**DOI:** 10.1002/ece3.8400

**Published:** 2021-12-01

**Authors:** Hajar El Hamss, Saptarshi Ghosh, M. N. Maruthi, Hélène Delatte, John Colvin

**Affiliations:** ^1^ Natural Resources Institute University of Greenwich Kent UK; ^2^ CIRAD, UMR PVBMT La Reunion France; ^3^ Department of Entomology University of Georgia Griffin Georgia USA

**Keywords:** and 16s rDNA, *Arsenophonus*, cross, SSA1, SSA1‐SG3, whitefly

## Abstract

A minimum of 13 diverse whitefly species belonging to the *Bemisia tabaci* (*B. tabaci*) species complex are known to infest cassava crops in sub‐Saharan Africa (SSA), designated as SSA1‐13. Of these, the SSA1 and SSA2 are the predominant species colonizing cassava crops in East Africa. The SSA species of *B. tabaci* harbor diverse bacterial endosymbionts, many of which are known to manipulate insect reproduction. One such symbiont, *Arsenophonus*, is known to drive its spread by inducing reproductive incompatibility in its insect host and are abundant in SSA species of *B. tabaci*. However, whether *Arsenophonus* affects the reproduction of SSA species is unknown. In this study, we investigated both the reproductive compatibility between *Arsenophonus* infected and uninfected whiteflies by inter‐/intraspecific crossing experiments involving the sub‐group three haplotypes of the SSA1 (SSA1‐SG3), SSA2 species, and their microbial diversity. The number of eggs, nymphs, progenies produced, hatching rate, and survival rate were recorded for each cross. In intra‐specific crossing trials, both male and female progenies were produced and thus demonstrated no reproductive incompatibility. However, the total number of eggs laid, nymphs hatched, and the emerged females were low in the intra‐species crosses of SSA1‐SG3A+, indicating the negative effect of *Arsenophonus* on whitefly fitness. In contrast, the inter‐species crosses between the SSA1‐SG3 and SSA2 produced no female progeny and thus demonstrated reproductive incompatibility. The relative frequency of other bacteria colonizing the whiteflies was also investigated using Illumina sequencing of 16S rDNA and diversity indices were recorded. Overall, SSA1‐SG3 and SSA2 harbored high microbial diversity with more than 137 bacteria discovered. These results described for the first time the microbiome diversity and the reproductive behaviors of intra‐/inter‐species of *Arsenophonus* in whitefly reproduction, which is crucial for understanding the invasion abilities of cassava whiteflies.

## Graphical abstract

The invasive SSA species of *B. tabaci* harbor diverse bacterial endosymbionts, many of which are known to manipulate insect reproduction. One such symbiont, *Arsenophonus*, is known to drive its spread by inducing reproductive incompatibility in its insect host and are abundant in SSA species of *B. tabaci*. This study showed that *Arsenophonus* decreased whitefly fitness and it is discussed with regard to whitefly microbiome.

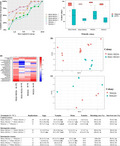

## 1 INTRODUCTION

The whitefly, *Bemisia tabaci* (*B. tabaci*), species is a complex of more than 40 morphologically indistinguishable species. At least 13 species of *B. tabaci* colonize cassava crops in sub‐Saharan Africa (SSA). Whiteflies are known to be infected with a primary endosymbiotic bacteria *Portiera*, and seven secondary endosymbionts (S‐endosymbionts): (i) *Cardinium*, (ii) *Arsenophonus*, (iii) *Hamiltonella*, (iv) *Rickettsia*, (v) *Wolbachia*, (vi) *Fritschea*, and (vii) *Hemipteriphilus aquaticus* (Bing et al., 2012; Chiel et al., 2007; Everett et al., 2005; Gottlieb et al., 2008). Of these, *Arsenophonus*, *Cardinium*, *Hamiltonella*, *Rickettsia*, and *Wolbachia* were prevalent in SSA1 whiteflies with *Arsenophonus* infection reaching up to 46.5% in Nigeria with three strains found (Akintola et al., 2020), while in East Africa, *Arsenophonus* infection reached 64%.

Most of these S‐endosymbionts are transmitted both vertically and horizontally (Bing et al., 2012; Gueguen et al., 2010; Marubayashi et al., 2014), and play several roles in *B. tabaci* biology such as providing higher fitness, protecting the insect from predatory wasps (Mahadav et al., 2008), mitigating heat stress (Brumin et al., 2011; Shan et al., 2014), increasing susceptibility to insecticides (Ghanim & Kontsedalov, 2009), and influencing reproduction by inducing cytoplasmic incompatibility (CI) (Hu & Li, 2015). Dual infection by *Arsenophonus* and *Rickettsia* decreased SSA1‐SG3 fitness compared to whiteflies without these bacteria (Ghosh et al., 2018).

The reproductive incompatibility (RI) between the whitefly *B. tabaci* species complex can be grouped into three categories (Liu et al., 2012; Qin et al., 2016; Sun et al., 2011). The first category, called prezygotic barrier, is characterized by a complete RI or mating barrier. In this case, two whitefly populations cannot mate with each other as courtship cannot occur. In the second category, called post‐zygotic barrier, inter‐breeding usually occurs but produces non‐fertile females. The last category is when whitefly populations can inter‐breed and produce viable offspring, characterized by successful gene flow. While some of the mechanisms behind these barriers are not fully understood, some S‐endosymbionts were shown to trigger the RI in some of *B. tabaci* species (Hu & Li, 2015). In particular, the S‐endosymbionts that can induce RI are also known as reproductive parasites or “master manipulators” and are prevalent in many whitefly species. *Arsenophonus nasoniae* is one of the master manipulators. In the wasp, *Nasonia vitripennis*, *A*. *nasoniae*, blocks 80% of the unfertilized eggs from developing into viable offspring and caused the death of offspring (Gherna et al., 1991). Other *Arsenophonus* spp. are also distributed among a variety of insects including whiteflies, aphids, psyllids, and a louse fly (Baumann, 2005; Dale et al., 2006), but their exact role in RI within African cassava whitefly species has been unknown. In this study, we investigated the role of *Arsenophonus* in whitefly reproduction and their population development which can lead to outbreaks.

Previous mating studies within and between *B. tabaci* have been generally related to *mtCOI* divergence (Qin et al., 2016), or whole‐genome single nucleotide polymorphisms (SNPs) and the full mitogenomes (Mugerwa et al., 2020), geography (Maruthi et al., 2004), and host plant adaptation (Burban et al., 1992), or infections by *Wolbachia* (Hu & Li, 2015) and *Cardinium* (Fang et al., 2014), but not *Arsenophonus*. Crossing experiments in relation to *Arsenophonus* infection will clarify its possible role in inducing RI or sex distortion which is crucial for understanding the invasion abilities of cassava whiteflies. We used isofemale lines of sub‐group three haplotypes of SSA1 (SSA1‐SG3) and SSA2 species with/without *Arsenophonus* to investigate the role of *Arsenophonus* in inducing the RI. The diversity of bacteria infecting the crossed parents and their progeny was further investigated by sequencing 16S rDNA.

## 2 MATERIALS AND METHODS

### 2.1 Whitefly colonies used in the crosses

Whitefly colonies with similar genetic background but differing only by *Arsenophonus* infection status were developed in these experiments and belonged to the sub‐group 3 haplotypes of the SSA1 (SSA1‐SG3A+ and SSA1‐SG3A−) and SSA2 (SSA2A+ and SSA2A−) species (accession numbers KM377902 and KM407142) (Ghosh et al., 2015). These two colonies were prepared from isofemale lines which have been maintained in NRI insectary for 20 years. Briefly, one female and two males were collected from core field colony and enclosed on an eggplant for 7 days to mate and oviposit. Parents were screened for their sequenced mtCO1 marker and endosymbiont composition as described in Ghosh et al. (2015). Both populations were confirmed to be free of all other known symbionts infecting *B. tabaci* using Illumina Hiseq sequencing of 16s rDNA marker. SSA1‐SG3 was originally collected from Tanzania, while SSA2 was collected from Uganda and reared in controlled conditions at NRI, University of Greenwich, UK. The purity of the whitefly colonies was assessed by PCR amplification of *mtCO1* and RFLP (Ghosh et al., 2015). All experiments were conducted on 2‐month‐old eggplants at about 27°C ± 2°C, 70% relative humidity, and photoperiod LD 12:12 h.

### 2.2 Preparation of plant material

Eggplants seeds (Var. Black Beauty) were planted singly in a medium‐size pot (4 cm height), which has an equal mixture of manure and organic soil. Eggplants pots were then covered and incubated in a pest‐free room for 2 months until reaching 6–7 fully expanded leaf stage. Plants were subsequently transferred into a pest‐free room and 2–3 were enclosed in whitefly‐proof cages with anti‐thrips and anti‐mites mesh. Before introducing eggplants into the cages, all leaves from each plant were carefully examined using a 20× magnification hand lens to ensure that only insect‐free plants were used.

### 2.3 Collection of emerged virgin whitefly adults

Three eggplants that reached the 5–6 leaf stage were introduced into core cages. These plants were monitored for 20 days to precisely determine the best time to initiate the experiment with day 0 corresponds to the day when the plant was introduced in the core cage and day 20 is the day post‐egg‐laying. To fetch nymphs that reached the late instar characterized by red eyes, leaves were monitored twice a day. Leaves with red‐eye pupae were then cut out. Small squares enclosing the pupae were also cut out from the eggplant leaves from each whitefly colony. Between 200 and 300 red‐eye pupae, nymphs were cut out from each colony and placed individually in glass tubes with wet cotton wool inside the boxes to increase humidity (Figure S1). The emerged adults were sexed under a binocular microscope before they were used in the crossing experiments.

### 2.4 Protocol adopted for crossing experiments

Three females and nine males were used in each replicate in the crossing experiments to provide multiple choices for mating. Both intra‐ and inter‐species crosses were conducted in this study. Intra‐specific crosses included SSA *B. tabaci* population from the same species but with different *Arsenophonus* infection status (e.g., SSA1‐SG3A+ × SSA1‐SG3A−). Intra‐species crosses were adopted to investigate the effect of *Arsenophonus* on (i) whitefly mating compatibility, (ii) hatching rate of eggs, and (iii) nymphs’ survival and female ratio within the same species. Inter‐specific crosses involved crosses with a different species but infected with *Arsenophonus* (SSA1‐SG3A+ × SSA2A+). These were carried out to test the effect of *Arsenophonus* on the same parameters but between different species.

For intra‐species, two reciprocal crosses (SSA1‐SG3A+ ♀ × SSA1‐SG3A− ♂ and SSA1‐SG3A− ♀ × SSA1‐SG3A+ ♂) were conducted using LLP containing one young eggplant with 1–2 leaves (Figure S1). Newly emerged adults were sexed under the microscope and three females and nine males were introduced into the LLP in the mornings. Seven days after the introduction, adults were collected back using a glass tube (Figure S1) and stored at −20℃ for later confirmation of bacterial infection. All eggs or nymphs produced were counted. After 30 days, the emerged F1 progenies were collected by opening the LLP in an empty whitefly‐proof cage. Empty pupal cases or remaining nymphs on eggplant leaves were also counted. The male‐to‐female ratio was calculated for each cross (Figure S1).

### 2.5 Whitefly DNA extraction for screening for bacteria in *B. tabaci*

DNA was extracted from a total of 29 whitefly samples using the Chelex method (Ghosh et al., 2015). The variable region in V4–V5 from 16s rDNA gene was then amplified by PCR using the primers F‐GTGCCAGCMGCCGCGG and R‐CCGTCAATTCMTTTRAGTTT (Wang et al., 2019), which were tagged with 12–13 bp unique barcodes (Table S1).

PCR amplifications were carried out in triplicates in a final volume of 25 µl using a Veriti thermocycler (Applied Biosystems, UK). Each reaction contained 3 µl of DNA template, 1x reaction buffer, 2.5 µM each primer, 10 mM dNTPs, and 1.0 U of Dream Taq DNA polymerase (Thermo Scientific, UK). The PCR conditions were an initial denaturation at 95°C for 2 min, 35 cycles of denaturation at 95°C for 30 s, annealing at 52°C for 15s, and extension at 72°C for 50 s; and a final extension at 72°C for 5 min. Triplicates of each sample were pooled before purification using a gel extraction kit (NucleoSpin, Macherey‐Nagel, Switzerland) according to the manufacturer's instructions and quantified with picogreen DNA quantification assay kit (Thermo Scientific, UK) in a qPCR machine (Biorad, CFX96, UK). Subsequently, amplicons of 410 bp obtained from each sample were pooled in equimolar concentrations in one centrifuge tube for Illumina HiSeq sequencing (FASTERIS SA, Switzerland). A composite sample with this pool of combined equimolar ratios was also subjected to a spin column purification using the same kit then quantification of the pool using Nanodrop. Another pool prepared in another centrifuge tube with the same samples was also sequenced in a different lane, but in the same flow cell to increase the depth of sequencing and the coverage of samples.

### 2.6 16S rDNA sequencing

The two pools with unique sequence tags contained 29 samples which were taken from different replicates of SSA1‐SG3A+, SSA2A+, and SSA2A−. *Escherichia coli* pure culture was included in each pool as a positive control to quantify the noise introduced during PCR and sequencing, and its potential contribution to the observed and estimated diversity. Quality filtering, chimera identification, and merging of paired‐end reads were carried out with the DADA2 plugin (Callahan et al., 2016). SILVA release 132 (Ref NR 99) (Quast et al., 2013) and VSEARCH consensus taxonomy classifier (Rognes et al., 2016) were simultaneously used of both lanes for classification of the 16S rDNA reads. Subsequently, the sequences were then clustered into groups called “Operational Taxonomic Units” (OTUs) based on 97% of similarity between them. Sequences classified as chloroplasts, *Portiera*, mitochondria were discarded from the analysis. Similarly, reads for which significant hits with known taxon could not be found were marked as unassigned. Another filtering step included the correction of 6 OTUs of the positive control (*E. coli*). Lastly, all the reads below 100 were also eliminated to minimize Illumina sequencing errors. Data filtering and statistical analysis were completed using R (R Core Team, 2017).

### 2.7 Data analysis

Whitefly adult emergence time distributions were compared using Anderson–Darling test. The proportion of eggs, nymphs, sex ratio, hatching rate, and survival rate was compared between the experimental and control crosses using a MANOVA test against treatment and then Tukey test per variable. To investigate the differences in bacterial diversity and communities within and between SSA1‐SG3 and SSA2, the colonies SSA1‐SG3A−, SSA1‐SG3A+, SSA2A+, and SSA2A− were filtered out from *Arsenophonus*. One‐way ANOVA test was also carried out to investigate bacterial diversity parameters such as Simpson index and observed OTUs. These were screened to investigate bacterial diversity across *B. tabaci* species generated from isofemale lines. Bray–Curtis dissimilarities between all pairwise combinations of whitefly samples were ordinated following a non‐metric multidimensional scaling (nMDS). The results of nMDS ordination were visualized on a scatter graph where the position of each whitefly sample depends on its distance from all other points in the analysis. This method reduced ecological community data complexity and identified meaningful relationships among the bacterial communities within SSA *B. tabaci*. Furthermore, metaMDS function, vegan (Oksanen et al., 2018), and ggplot2 libraries were used for data analysis and visualization (Ginestet, 2011).

## 3 RESULTS

### 3.1 Whitefly reproduction and survival rate

**FIGURE 1:**
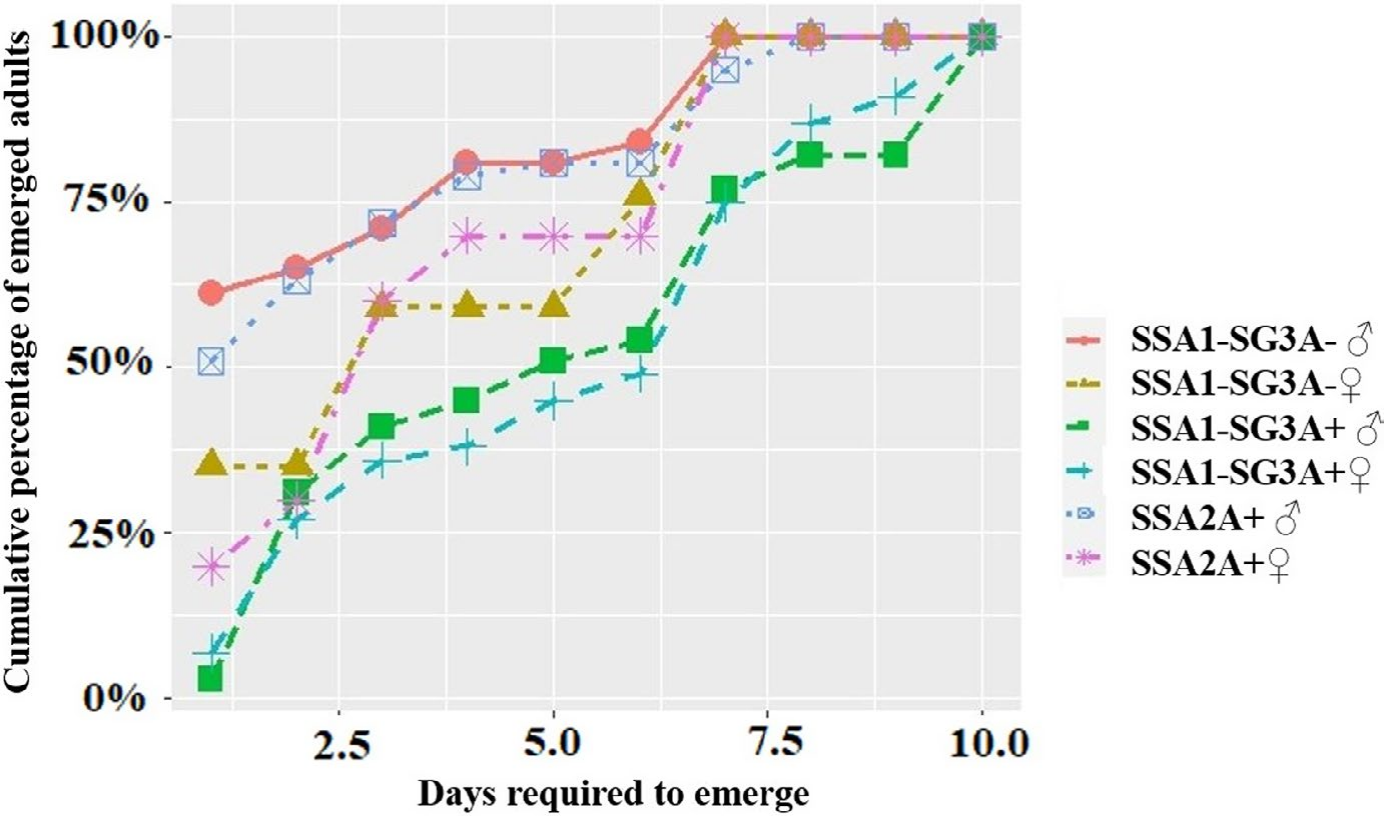
Comparison of cumulative percentage of emerged males and females through time between tested whitefly species. The starting day corresponds to the day when the red‐eye pupa was enclosed in the small glass tube attached to a small part of the eggplant leaf. Temperature was 27°C ± 2°C, 70% relative humidity, and photoperiod LD 12:12 h

Average number of eggs, nymphs, females, and males were significantly different between treatments based on MANOVA test (*p* = 9.817e‐14). There were significant differences in the average number of eggs laid with the highest average number recorded in the cross between SSA1‐SG3A− ♀ and SSA2A+ ♂ (86.2 ± 30.5) (Table 1). Similarly, recorded nymph numbers were the highest in the cross between SSA1‐SG3A− ♀ and SSA2A+ ♂ (60.4 ± 32) with 70% of hatching rate from egg to nymphs (Table 1). Average hatching rates were the highest in *Arsenophonus*‐free whiteflies (SSA1‐SG3A−) reaching 90% (Figure 1). Average survival rate from nymphs to adults was high in most crosses reaching 90%, but very low in the inter‐species cross where only 20% of adults survived (Table 1).

**TABLE 1:** Fitness parameters of F1 whiteflies generated from the various crosses. Three females and nine males were used per cross

Treatment (3♀*9♂)	Replications	Eggs	Nymphs	Males	Females	Hatching rate (%)	Survival rate (%)
Control crosses							
SSA1‐SG3A+♀*SSA1‐SG3A+**♂**	26	41.5 ± 29.3 ac	29.7 ± 24 abc	11.5 ± 15.6 a	3.6 ± 5.6 a	80 ± 0.3 b	70 ± 0.3 bc
SSA1‐SG3A−♀*SSA1‐SG3A−**♂**	21	58.5 ± 30.6 bc	53.5 ± 33.8 bd	17.9 ± 12.7 abc	17.7 ± 22.4 b	90 ± 0.3 b	90 ± 0.2 b
SSA2A+♀*SSA2A+**♂**	9	30.7 ± 17.4 ac	30.7 ± 17.4 abcd	12.1 ± 8.5 ab	4.4 ± 3.8 ab	100 ± 0 b	50 ± 0.2 ac
Intra‐species crosses							
SSA1‐SG3A+♀*SSA1‐SG3A−**♂**	25	31.3 ± 25.9 a	26.2 ± 26.6 ac	12.2 ± 12.1 a	2.7 ± 3.7 a	80 ± 0.3 b	90 ± 0.2 b
SSA1‐SG3A−♀*SSA1‐SG3A+**♂**	11	52 ± 31 abc	49.1 ± 29.7 bcd	33.7 ± 22 c	18.4 ± 25.5 b	100 ± 0.1 b	90 ± 0.2 b
Inter‐species crosses							
SSA1‐SG3A+♀*SSA2A+**♂**	6	26.2 ± 13.2 ac	26.2 ± 13.2 abcd	9 ± 5.2 ab	0 ± 0	100 ± 0 b	40 ± 0.2 ac
SSA2A+♀*SSA1‐SG3A+**♂**	5	14.8 ± 6.6 a	14.8 ± 6.6 abc	11.2 ± 7.3 abc	0 ± 0	100 ± 0 b	100 ± 0.1 b
SSA1‐SG3A−♀*SSA2A+**♂**	12	86.2 ± 30.5 b	60.4 ± 32 d	29.2 ± 21 bc	0 ± 0	70 ± 0.3 b	90 ± 0.3 b
SSA2A+♀*SSA1‐SG3A−**♂**	8	20.6 ± 13.2 a	5.5 ± 5.9 a	1.4 ± 1.9 a	0 ± 0	30 ± 0.4 a	20 ± 0.3 a

Note

Means followed by the same letters are not significantly different, while means followed by different letters are significantly different as separated by Tukey's HSD test at *p* < .05.

### 3.2 Adult emergence and proportion of females

Emergence time of males was significantly shorter for SSA1‐SG3A− compared to SSA1‐SG3A+ whiteflies (Anderson–Darling test, *p* = .03; Figure 1). Emergence time of females of both SSA1‐SG3A+ and SSA1‐SG3A− was not statistically different (*p* = .54; Figure 1). In addition, emergence time of both SSA1‐SG3A+ and SSA2A+ males and females was not statistically different (*p* = .41).

*Arsenophonus* did not induce reproductive incompatibility within SSA1‐SG3 population as females were produced in both controls and treatments. However, there were significant differences in the proportion of emerged female adults for the different crosses. A higher proportion of females were recorded in crosses involving SSA1‐SG3A− (17.7 ± 22.4) than SSA1‐SG3A+ (3.6 ± 5.6). The cross SSA1‐SG3A+ ♀ * SSA1‐SG3A− ♂ had a lower proportion of average females with only 2.7 ± 3.7. In contrast, *Arsenophonus* induced reproductive incompatibility between SSA1‐SG3 and SSA2 as no females were produced in any combination involving these two species (Table 1).

### 3.3 Confirmation of the status of *Arsenophonus*

Over 9 million (9,325,004) clean reads were generated from Illumina HiSeq platform for the 29 whitefly samples. After filtering both low‐quality sequences and those that belonged to *Portiera*, chloroplast, and mitochondria, a total of 7,639,071 were assigned to the bacterial community and 1,685,933 reads were assigned to S‐endosymbionts. The overall recovered clean reads were different for each *B. tabaci* species (Table S2), with an average read of 282,576 obtained per sample with an average sequence length of 377 bp. Finally, a total of 80,765 reads were unassigned to any of the previously known OTUs from Silva database.

The presence of *Arsenophonus* and other bacteria was examined in parents and progeny of the species SSA2A+, SSA1‐SG3A+, and SSA1‐SG3A−. *Arsenophonus* reads in parents of SSA1‐SG3A+ and SSA1‐SG3A− were as expected, 112,754 and 0, respectively (Table S2), whereas the progeny from the cross SSA1‐SG3A+ and SSA1‐SG3A− had 158,244 and 0 reads, respectively. *Arsenophonus* reads from SSA2A+ parents reached 163,251 (Table S2). All these populations were found to be free from known S‐endosymbionts.

### 3.4 Microbiome diversity within and between SSA cassava *B. tabaci*

**FIGURE 2:**
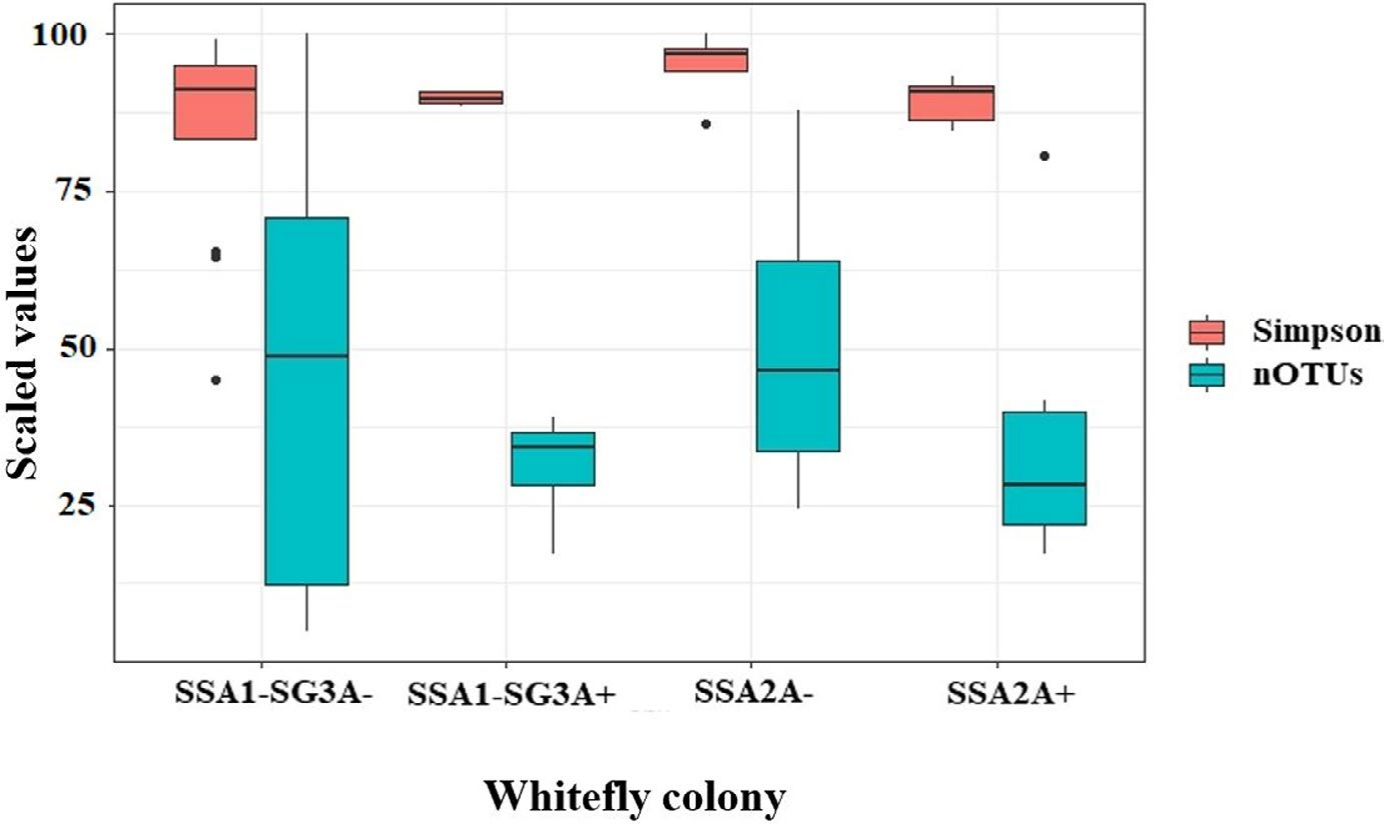
Alpha diversity analysis for each SSA *Bemisia tabaci* species using Simpson indices and the number of OTUs

**FIGURE 3:**
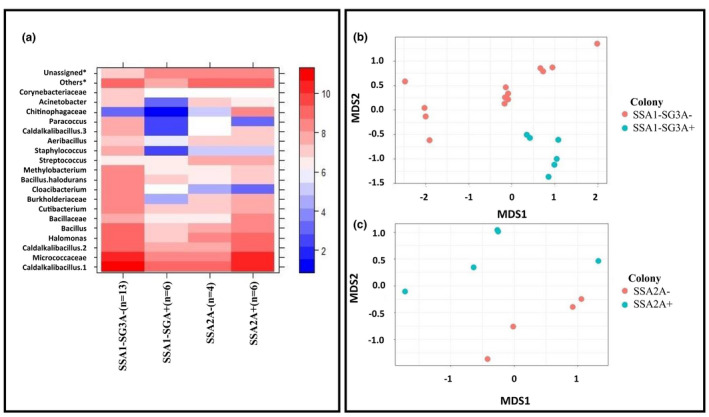
Bacterial composition (a, b and c) detected in whitefly colonies. Heat map of log‐reads of most abundant bacteria detected in whitefly colonies including those used in the crossing trials (a). Some of them were identified at the family level, whilst the majority were at the genus level (a). Bacterial composition was shown in nMDS graph for SSA1‐SG3 (b) and SSA2 (c). *others = all non‐abundant OTUs aggregated together, Unassigned = all OTUs not assigned with any OTU in the database

Apart from *Arsenophonus*, several sequences of “other bacteria” were also detected in SSA *B. tabaci* species. Both parents from SSA1‐SG3A+ and SSA1‐SG3A− had 19,592 and 64,804 reads assigned to other bacteria (Table S2). The progeny from SSA1‐SG3A+ and SSA1‐SG3A− also had sequence reads of up to 68,728 and 291,754 reads, respectively, assigned to other bacteria (Table S2). In SSA2A+, relatively lower reads of 33,846 were assigned to other bacteria (Table S2 and S3). Simpson (*p* = .44) and the number of OTUs were similar in all tested whiteflies (*p* = .35, Figure 2), whereas Bray–Curtis matrix showed significant differences (*p* = .008, Figure 3b). SSA2A+ and SSA2A‐ also vary significantly (*p* = .019, Figure 3c). Heat map of bacterial differences showed nine different OTUs including *Bacillaceae*, *Burkholderiaceae*, *Cloacibacterium*, *Methylobacterium*, *Staphylococcus*, *Caldalkalibacillus*‐3, *Paracoccus*, *Acinetobacter*, and *Corynebacteriaceae*. Those OTUs were prevalent in SSA1‐SG3A− but absent from SSA1‐SG3A+ (Figure S2 and Figure 3a). A different trend was shown between SSA2A+ and SSA2A‐ with SSA2A+ having *Bacillaceae*, *Methylobacterium*, and *Caldalkalibacillus*‐3 but SSA2A‐ did not harbor these OTUs, indicating that SSA1‐SG3A− is compensating for the loss of *Arsenophonus* by harboring other OTUs but this is species dependent.

## 4 DISCUSSION

*Bemisia tabaci* species harbor multiple reproductive manipulators. Identifying them and their relative frequency with other extracellular gut symbionts is essential in understanding the reproduction of this pest.

Before discussing these results, we highlight that we were unsuccessful in generating SSA2A‐ colony without *Arsenophonus* as these whiteflies failed to produce a viable progeny after several attempts. Previous experiments involving the use of antibiotics have also not been able to completely eliminate *Arsenophonus* in singly infected whiteflies (Wang et al., 2020), or when co‐infected with both *Arsenophonus* and *Rickettsia* (Ghosh et al., 2018). One study successfully eliminated 100% of *Arsenophonus* but failed to completely eliminate other infections such as *Wolbachia* and *Rickettsia*. The removal was only 39% for *Wolbachia* and 27% for *Rickettsia* (Wang et al., 2020). Nevertheless, this study constitutes the first attempt to investigate the induction of reproductive incompatibility induced by *Arsenophonus* in two different whitefly species SSA1‐SG3 and SSA2.

In this study, we conducted both intra‐ and inter‐species crosses, using SSA1‐SG3 and SSA2 species differing in *Arsenophonus* infections. We also investigated in‐depth bacterial diversity of those crossed parents and their progeny which revealed that *Arsenophonus* was the only S‐endosymbiont present in the crossed colonies. In SSA1‐SG3A−, *Arsenophonus* was absent from all samples.

In intra‐species crosses, no sign of RI was observed as females were produced equally in both controls and treatment crosses. However, a reduction in eggs, nymphs, and females was recorded between two controls of SSA1‐SG3 infected with *Arsenophonus*, which is indicating that *Arsenophonus* negatively impacted SSA1‐SG3 fitness. *Arsenophonus* infection thus decreased fitness of this whitefly species, a similar result was obtained in an earlier study from the same species (Ghosh et al., 2018). Nevertheless, on other *B. tabaci* species, such as the Asia II, *Arsenophonus* did not have any effect on the progeny (Raina et al., 2015). Those discrepancies could be linked to several factors, such as the different genetic background of the host or the differences between strains of *Arsenophonus*. Indeed, different strains of *Arsenophonus* had been described in several *B. tabaci* species: (i) Asia II 3, (ii) Asia II 7, (iii) Indian Ocean, (iv) MED, (v) Asia II 1, and (vi) Asia I and were linked to beneficial, neutral, or harmful effects depending on the *B. tabaci* species (Ahmed et al., 2009; Chiel et al., 2007; Gueguen et al., 2010; Singh et al., 2012; Thierry et al., 2011).

*Arsenophonus* has also been involved in enhancing virus transmission capacity within their *B. tabaci* vector (Alberto Bressan et al., 2007; Danet et al., 2003; Zreik et al., 1998) (Rana et al., 2012). However, in laboratory conditions, the concentration of *Arsenophonus* increased significantly in whiteflies feeding on tomato plants infected with *Tomato leaf curl Bangalore virus* (ToLCBV) (Prasannakumar & Maruthi, 2021). It is a prevalent S‐endosymbiont in SSA whiteflies cassava pandemic regions affected by cassava mosaic disease (CMD), suggesting their potential role in CMD transmission. Similar association between *Arsenophonus* and CMD was observed in India (Harish et al., 2019).

In inter‐species crosses, no females were produced. These results thus showed complete RI and lack of gene flow between these populations. To confirm this further, experiments are required with SSA2A‐ as this colony failed to develop in this study. Similarly, in a recent crossing experiment, SSA1‐SG3 and SSA2 were unable to produce females (Mugerwa et al., 2020).

Reduction in survival rate is caused by disruption of nymph development. This was shown with *Arsenophonus* in parasitic wasps which caused reproductive manipulation by killing male progeny (also called a son‐killing factor) (Gherna et al., 1991; Nadal‐Jimenez et al., 2019; Skinner, 1985; Werren et al., 1986). In reciprocal cross involving SSA2A+ and SSA1‐SG3A+, almost 80% of eggs did not hatch, demonstrating mating barriers between these two species. Inter‐species mating without effective eggs or nymphs hatching is a partial RI where courtship occurs but females are not produced (Gröning & Hochkirch, 2008).

The 16s rDNA sequencing revealed a total of 137 valid taxa and about 25 OTUs per sample. In previous studies, an average of 3, 5, and 6 OTUs from MEAM1, MED, and Asia I, respectively, were discovered (Jing et al., 2014). The discrepancy between these studies could be due to lack of standard protocols used for bacterial diversity studies. Other factors such as (i) different coverage, (ii) platform, and (iii) library preparation could also contribute to such variations, making it difficult to compare different studies. In this study, other bacteria were detected in SSA whiteflies, which were reared in laboratory conditions for a long time. In this study, we detected *Paracoccus* and *Acinetobacter* which were previously detected in Asia I and Asia II whiteflies (Singh et al., 2012). Also, *Staphylococcus* was detected in both MED and MEAM1 species (Indiragandhi et al., 2010). Similarly, Asia I and Asia II5 from India harbored *Bacillus*, *Enterococcus*, and *Bacteroide* (Harish et al., 2019).

Other bacteria which were also detected in this study were previously reported in the gut of other insect species. For example, species belonging to *Bacillaceae*, *Burkholderiaceae*, *Acinetobacter*, *Cloacibacterium*, and *Staphylococcus* were abundant in the mid‐gut of tsetse flies (Glossina sp.) (Griffith et al., 2018), *Corynebacteriaceae* in scabies (*Sarcoptes scabiei*) (Swe et al., 2019), *Methylobacterium* in the mosquito (*Aedes aegypti*) (Muturi et al., 2021), *Burkholderiaceae* in both *Tetraponera ants nd in aphids* (*Myzus persicae*) (He et al., 2021), and *Bacillaceae* in the gut of the melon fruit fly (*Bactrocera cucurbitae*) (Mishra et al., 2018). To our knowledge, *Caldalkalibacillus*, which is a species belonging to *Bacillus*, was detected first time in whiteflies. Other techniques such as fluorescent in situ hybridization (FISH) should be used to confirm the presence and location of the many bacteria found in African cassava whiteflies.

The mating behavior of arthropods is regulated by bacteria which are located in their reproductive organs (Jordan & Tomberlin, 2021). Small differences in these bacteria composition can prevent mating within insects (Otti, 2015). Some bacteria can enter through mating wounds and contaminate reproductive organs or even enter the body cavity (Otti, 2015). Little is known about the microbial composition in reproductive organs of whiteflies. Identification and localization of bacteria which are present in whitefly reproductive organs can help understand the bacterial effect on whitefly reproduction and development.

In summary, we found that (i) *Arsenophonus* did not induce reproductive incompatibility within SSA1‐SG3 but reduced the number of eggs, nymphs, and female ratios, (ii) complete RI was observed between SSA1‐SG3 and SSA2 indicating the lack of gene flow between the two whitefly species, and (iii) many new “other bacteria” in SSA *B. tabaci* have been identified, whose role remains to be investigated.

## CONFLICT OF INTEREST

The authors declare no conflict of interest.

## AUTHOR CONTRIBUTIONS

**Hajar El Hamss:** Data curation (lead); Formal analysis (lead); Methodology (lead); Software (lead); Writing – original draft (lead). **Saptarshi Ghosh:** Methodology (equal); Writing – review & editing (equal). **Hélène Delatte:** Conceptualization (equal); Funding acquisition (equal); Investigation (equal); Methodology (equal); Resources (equal); Supervision (equal); Validation (lead); Visualization (equal); Writing – review & editing (lead). **M. N. Maruthi:** Conceptualization (lead); Funding acquisition (equal); Investigation (equal); Project administration (equal); Resources (equal); Supervision (lead); Validation (equal); Visualization (equal); Writing – review & editing (equal). **John Colvin:** Conceptualization (equal); Funding acquisition (lead); Investigation (equal); Project administration (equal); Supervision (equal).

## Supporting information

Supplementary Material

## Data Availability

The raw data used to estimate symbionts frequency and composition, as well as the sample information (Figures 1, 2, 3; Figure S2 and Table 1; Tables S2 and S3), were deposited to Dryad at the following URL:
OTU data and sample information files: https://doi.org/10.5061/dryad.xsj3tx9gc. Crossing experiment data: https://doi.org/10.5061/dryad.xsj3tx9gc. Script used for statistical analysis: DOI https://doi.org/10.5061/dryad.xsj3tx9gc (used to construct all Figures). [Corrections added on 20 December 2021, after first online publication: In the Data Availability Statement, the dryad links have been updated.]
