# Supplementary material for: Microbiome diversity and reproductive incompatibility induced by the prevalent endosymbiont Arsenophonus in two species of African cassava Bemisia tabaci whiteflies
Source: Ecol Evol. 2021 Dec 1;11(24):18032–41. doi: 10.1002/ece3.8400 (PMC8717322; doi:10.1002/ece3.8400)

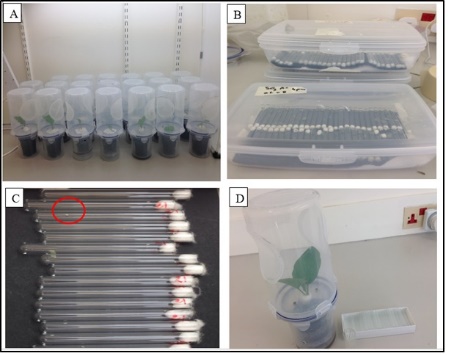


**Figure S1: Rearing unit used for crossing experiments and cohorts. A) Lock-lock pots (LLP) ready for setting up crossing experiments. B) Incubation of nymphs in glass tubes: Tubes with red-eye pupa attached to leaves. C) Emergence of adults in glass tubes. D) 3 females and 9 males transferred to the LLP to allow mating and egg laying for seven days.**

**Table S1: Barcode sequences used for sequencing the 16S rDNA of the whitefly endosymbionts.**

**
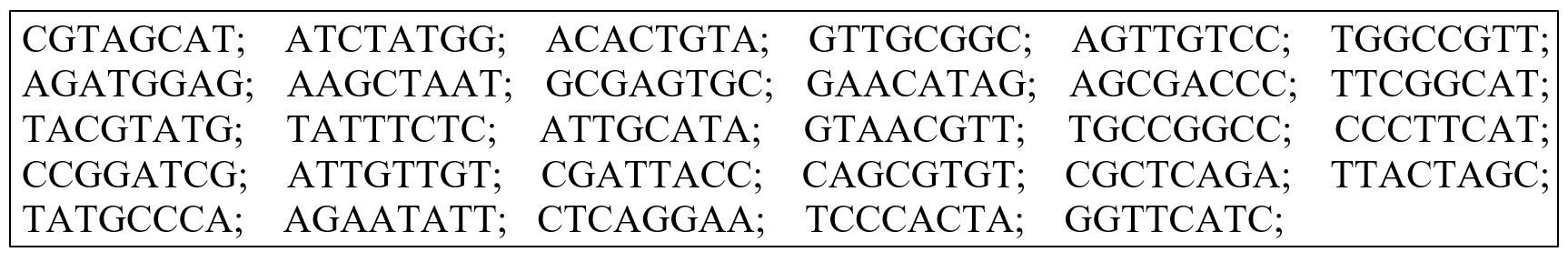
**

**Table S2: Overall recovered 16S rDNA reads of whitefly bacterial infections before and after filtering of two merged lanes.**


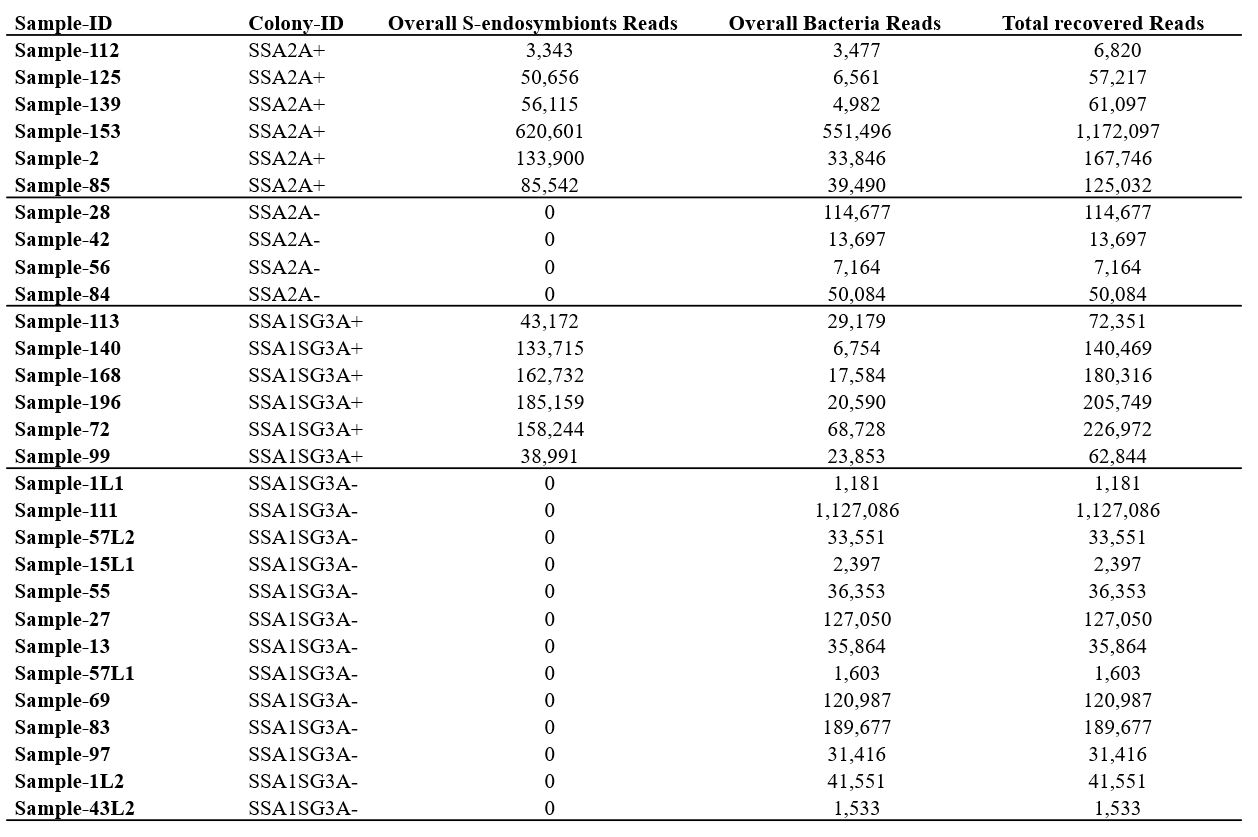


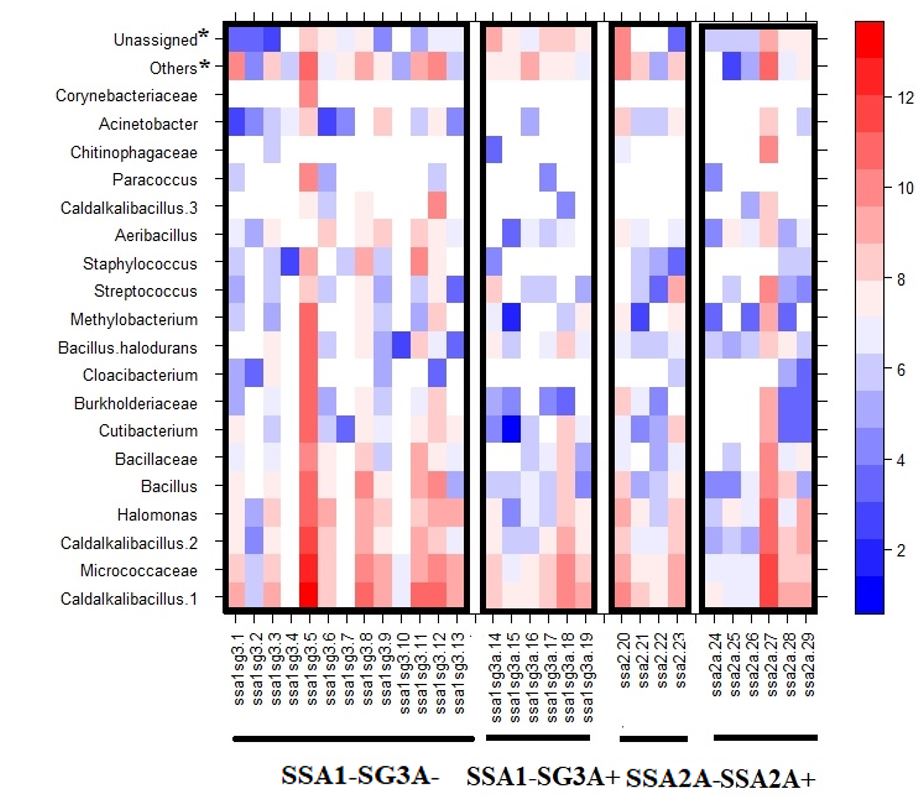


**Figure S2: Heatmap of most abundant OTUs per colony based on log of 16S rDNA reads.**

**Table S3: Average of 16S rDNA reads assigned to *Arsenophonus* and other bacteria in control crosses between SSA1-SG3 and SSA2.**


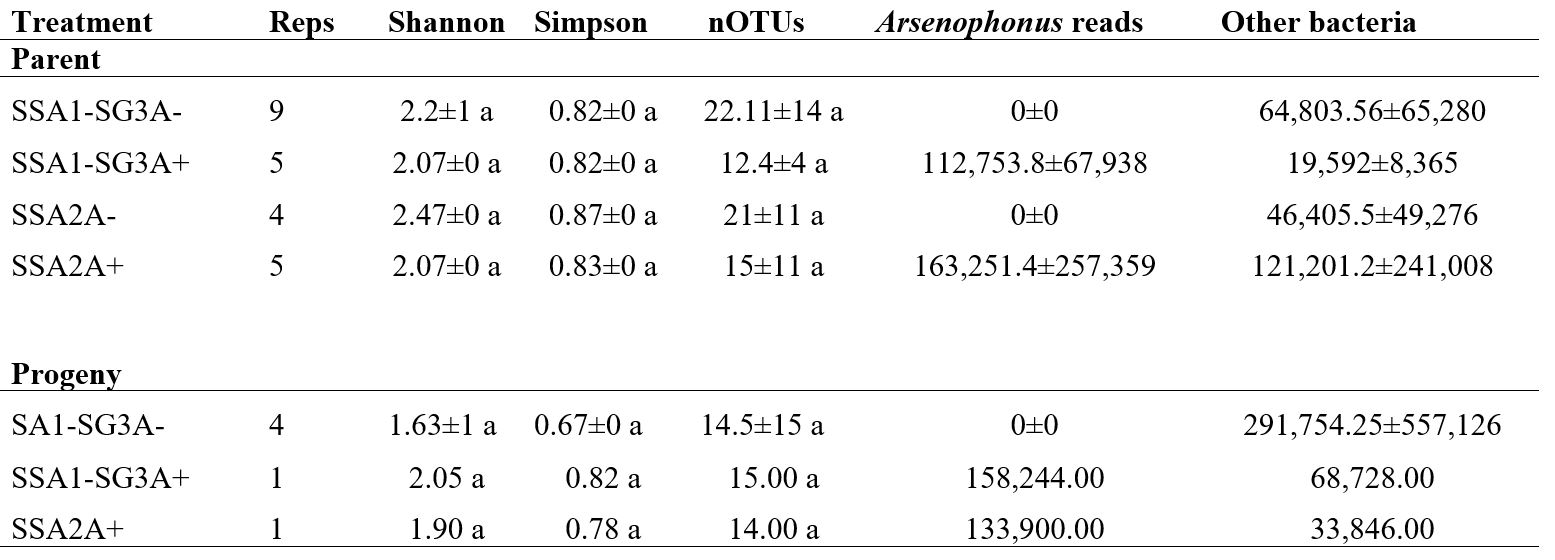

Supplement: Supplementary file 1 — Supplementary Material [file ECE3-11-18032-s001.docx]
